# Mapping the Prevalence and Risk Factors of Low Back Pain Among University Populations in Saudi Arabia: A Systematic Review and Meta-Analysis

**DOI:** 10.3390/jcm15072808

**Published:** 2026-04-07

**Authors:** Sulaiman Alanazi, Jana Alruwaili, Maysam Alruwaili, Abdulmajeed Alfayyadh, Hadeel Alsirhani, Samaher Mohammed Alowaydhah, Sultan A. Alanazi, Nesma M. Allam, Sara Elsebahy

**Affiliations:** 1 Department of Physical Therapy and Health Rehabilitation, College of Applied Medical Sciences, Jouf University, Sakaka 72346, Saudi Arabia; jana.al4@icloud.com (J.A.); mayasm2487@gmail.com (M.A.); abalfayyadh@ju.edu.sa (A.A.); hsalserhany@ju.edu.sa (H.A.); smalowaydhah@ju.edu.sa (S.M.A.); nmallam@ju.edu.sa (N.M.A.); 2 Department of Physical Therapy and Health Rehabilitation, College of Applied Medical Sciences, Majmaah University, Al-Majmaah 11952, Saudi Arabia; sa.alanazi@mu.edu.sa; 3 Department of Physical Therapy for Pediatrics Therapy, Faculty of Physical Therapy, Kafrelsheikh University, Kafrelsheikh 33511, Egypt; sarah_elsehy2014@pt.kfs.edu.eg

**Keywords:** low back pain, prevalence, risk factors, university students, faculty, ergonomics, Saudi Arabia

## Abstract

**Background/Objectives**: Low back pain (LBP) is one of the most common musculoskeletal conditions globally and a leading cause of disability. University populations may be particularly vulnerable due to prolonged sitting, academic stress, and frequently suboptimal ergonomics, especially in rapidly expanding higher education systems such as those in Saudi Arabia. This systematic review and meta-analysis aimed to synthesize evidence on the prevalence of LBP among university attendants in Saudi Arabia and to quantify its associations with key demographic and environmental risk factors. **Methods**: We systematically reviewed observational studies reporting LBP prevalence and/or risk factors among university students and faculty in Saudi Arabia published in English, following Cochrane methodological guidance and PRISMA 2020 reporting recommendations. The protocol was prospectively registered in PROSPERO (CRD420250654048). We searched PubMed, Embase and CINAHL from inception to February 2025. Two reviewers independently screened studies, extracted data, and assessed risk of bias using the Joanna Briggs Institute checklist for analytical cross-sectional studies. Random effects meta-analyses were used to pool prevalence estimates across recall periods, regions, populations, and measurement tools, and to calculate pooled odds ratios (ORs) for age, sex, smoking, family history of LBP, and college seating conditions. Heterogeneity, subgroup, and sensitivity analyses were undertaken. **Results**: Thirteen cross-sectional studies were included. The overall pooled prevalence of LBP was 57% (95% confidence interval [CI] approximately 43–71), with substantial heterogeneity. Prevalence varied by recall period, region, population group, and measurement instrument; pooled prevalence was 58% among students and 50% among faculty. Increasing age (OR 1.17, 95% CI 1.01–1.34) and poor college seating conditions (OR 1.42, 95% CI 1.07–1.76) were significantly associated with LBP. Male gender, smoking, and family history showed non-significant pooled effects. These estimates are limited by substantial between-study heterogeneity, variable measurement tools, and exclusively cross-sectional designs, which restrict causal inference. **Conclusions**: LBP is prevalent among university attendants in Saudi Arabia, affecting both students and faculty. The consistent associations with age and seating ergonomics highlight the need for ergonomic classroom redesign and age-sensitive preventive strategies. Future work should adopt standardized LBP measures and longitudinal designs to clarify causal pathways and evaluate targeted interventions. Funding: This work was supported by the Deanship of Graduate Studies and Scientific Research at Jouf University (grant DGSSR-2026-NF-01-002).

## 1. Introduction

Low back pain (LBP) is one of the most common musculoskeletal disorders globally and a leading cause of disability across high-, middle-, and low-income countries [1,2]. It is typically defined as pain localized between the 12th rib and the inferior gluteal folds, with or without leg pain, and in most cases no specific underlying pathology can be identified, leading to a diagnosis of non-specific LBP [3,4]. Recent global burden estimates indicate that, in 2019, there were approximately 568 million prevalent cases of LBP worldwide, with substantial associated years living with disability, affirming LBP as a major public health challenge [1]. In the Middle East and North Africa [2] region, including Saudi Arabia, the burden of LBP remains high despite modest declines in age-standardized prevalence over recent decades [2]. Within Saudi Arabia, population-based estimates suggest that LBP prevalence in adults ranges from roughly one-fifth to over one-half, underscoring its clinical and socioeconomic impact [5].

The etiology of LBP is multifactorial, reflecting interactions between biological, mechanical, and psychosocial determinants. Established and emerging risk factors include increasing age, female or male sex depending on context, obesity, poor physical fitness, psychosocial distress (e.g., stress, anxiety, depression), occupational and educational demands, smoking, and prolonged static postures [6,7,8]. Among younger adults, particularly those in educational settings, modifiable factors such as sedentary behavior, long hours of sitting, heavy backpack carriage, and suboptimal ergonomics (e.g., nonadjustable seating and desks) have been repeatedly implicated [6,9]. University students may therefore face a unique constellation of risk exposures—high academic load, extensive screen time, and often limited opportunities for physical activity—which can predispose them to both acute and recurrent LBP and may contribute to pain chronicity into later adulthood [1,9].

University attendants, including students and academic or administrative staff, represent a strategically important population from a rehabilitation and public health perspective. LBP in this group can adversely affect class attendance, concentration, physical functioning, and productivity, with downstream effects on educational attainment and work performance [6,9]. In Saudi Arabia, rapid expansion of the higher education sector and increasing enrolment in universities have created large, relatively young cohorts who may be exposed to prolonged sedentary behaviors and non-ergonomic learning environments. Recent single-center studies from Saudi universities report high point and period prevalence of LBP among college students and faculty, and they highlight associations with prolonged sitting, uncomfortable lecture hall seating, heavy lifting, and study-related stress [10,11]. These findings support the hypothesis that contextual factors specific to Saudi universities—such as classroom design, timetable structure, and cultural norms regarding physical activity—may contribute importantly to LBP risk.

Despite the growing body of primary research, no prior review has comprehensively synthesized the prevalence and risk factors of LBP specifically among university attendants in Saudi Arabia using systematic and meta-analytic methods. Previous reviews in the Saudi context have focused largely on healthcare workers or general adult populations, and international syntheses often aggregate heterogeneous educational settings across countries with markedly different ergonomics, curricula, and health systems [5,8]. Consequently, there remains uncertainty regarding the pooled prevalence of LBP across different time frames (e.g., point, 12 months, lifetime) and subgroups (students vs. faculty; regions of the country) as well as the strength and consistency of associations with key modifiable risk factors such as seating ergonomics, smoking, and psychosocial stressors. This evidence gap limits the ability of policymakers, university administrators, and rehabilitation professionals to design targeted, evidence-based interventions and to prioritize resource allocation for prevention and early management of LBP in higher education settings.

In accordance with Cochrane guidance for systematic reviews, this review was designed a priori with a clearly defined research question, protocol registration, explicit eligibility criteria, and a comprehensive search strategy to ensure transparency and reproducibility [12,13]. The primary objective was to estimate the pooled prevalence of LBP among university attendants in Saudi Arabia across different recall periods and population subgroups. The secondary objective was to identify and quantify associations between LBP and key risk factors reported in the literature, including age, gender, smoking, family history of LBP, and seating arrangements, using meta-analytic techniques where appropriate. By synthesizing and critically appraising the available evidence, this systematic review and meta-analysis aims to provide a robust epidemiological foundation for developing ergonomically informed, context-specific prevention and rehabilitation strategies within Saudi universities. In particular, this review provides the first systematic, meta-analytic comparison of LBP prevalence between students and faculty within Saudi universities, set against the backdrop of rapid expansion of the national higher education system.

## 2. Materials and Methods

### 2.1. Protocol and Registration

This systematic review and meta-analysis was conducted in accordance with the methodological guidance of the Cochrane Handbook for Systematic Reviews of Interventions and reported with reference to the PRISMA 2020 statement [13,14] (Files S1 and S2). The review protocol was developed a priori, specifying the research question, eligibility criteria, search strategy, and planned methods for data extraction and synthesis. The protocol was prospectively registered on the International Prospective Register of Systematic Reviews (PROSPERO) under registration number CRD420250654048. No amendments were made to the registered protocol after registration.

### 2.2. Eligibility Criteria

The inclusion and exclusion criteria were defined using the population, exposure, outcome, and study design framework, tailored to the epidemiological nature of the review.

#### 2.2.1. Population

University attendants in Saudi Arabia, including students, academic staff, and other employees working within university settings, of any sex, any nationality, and aged ≥18 years were eligible.

#### 2.2.2. Exposure/Setting

Attendance or employment in a Saudi Arabian university, regardless of discipline or faculty, was required.

#### 2.2.3. Outcomes

Primary outcome: quantitative estimates of low back pain (LBP) prevalence, defined as pain localized between the 12th rib and the inferior gluteal folds, with or without leg pain, over specified time frames (e.g., point, 1 week, 12 months, lifetime) [3,4]. Secondary outcomes: associations between LBP and potential risk factors, including age, sex, smoking, family history of LBP, and college seating/furniture characteristics, reported as odds ratios (ORs) or convertible effect measures.

#### 2.2.4. Study Design

Observational analytical and descriptive designs (primarily cross-sectional studies) reporting quantitative prevalence data and/or risk factor estimates were eligible.

#### 2.2.5. Language and Location

Studies conducted in Saudi Arabia and published in English were included.

#### 2.2.6. Exclusion Criteria

Non-human studies, qualitative designs, narrative reviews, systematic reviews, editorials, letters, commentaries, case reports, and conference abstracts without extractable quantitative data were excluded. Studies conducted outside Saudi Arabia or published in languages other than English were also excluded.

### 2.3. Information Sources and Search Strategy

A comprehensive electronic search was undertaken in three major databases, PubMed (MEDLINE), Embase, and CINAHL, from database inception to the latest search data specified in the protocol. The search strategy combined controlled vocabulary (e.g., MeSH terms in PubMed, Emtree in Embase) and free-text terms related to low back pain, Saudi Arabia, and university populations from conception to February 2025.

For PubMed and Embase, the core search string used combinations of terms such as:

(low back pain OR non-specific low back pain OR lumbago OR coccyx OR mechanical low back pain OR spondylosis OR backache OR lower back pain OR musculoskeletal pain) AND (Kingdom of Saudi Arabia OR Saudi Arabian OR KSA OR SA) AND (prevalence OR incidence) AND (university OR students OR workers OR class OR university attendees OR college).

Boolean operators (“AND”, “OR”) and truncation were applied to maximize sensitivity while maintaining specificity for the target population. Reference lists of all included articles and relevant reviews were hand-searched to identify additional eligible studies not captured by the electronic search. Where necessary, gray literature sources (e.g., institutional repositories) were screened to minimize publication bias. All databases were searched from inception to February 2025.

#### 2.3.1. Study Selection

All identified records were imported into Covidence, an online systematic review management platform, where duplicates were automatically detected and removed. Study selection proceeded in two stages.

#### 2.3.2. Title and Abstract Screening

Two reviewers independently screened titles and abstracts against the predefined inclusion and exclusion criteria. Records clearly not meeting the criteria were excluded at this stage.

#### 2.3.3. Full-Text Review

The full texts of potentially eligible articles were retrieved and independently assessed by the same two reviewers. Discrepancies at any stage were resolved through discussion; if consensus could not be reached, a third reviewer would be adjudicated.

**Figure 1:**
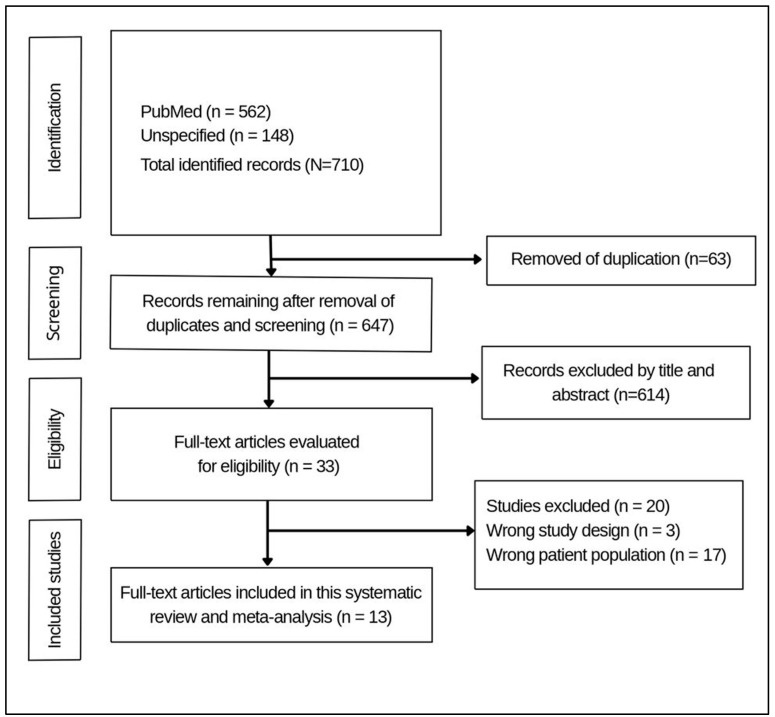
PRISMA flow diagram.

Reasons for exclusion at the full-text stage (e.g., wrong population, no quantitative prevalence data, not conducted in Saudi Arabia) were documented. The overall selection process is summarized in a PRISMA flow diagram (Figure 1), detailing the number of records identified, screened, included, and excluded.

#### 2.3.4. Data Extraction

Data extraction was conducted in Covidence using a standardized, piloted data extraction form developed for this review. Two reviewers independently extracted data from each included study, and disagreements were resolved by consensus or adjudication by a third reviewer.

The following variables were collected:

- Bibliographic details: First author, year of publication, journal.
- Study characteristics: Region within Saudi Arabia (Eastern, Western, Northern, Southern, Middle), study design, data collection period, sampling method, and response rate.
- Population characteristics: Sample size, age (mean, range), sex distribution, role within university (student, faculty, other staff), and discipline if reported.
- Outcome measurement: Definition of LBP used, recall period (e.g., point, 1 week, 12 months, lifetime, >3 months), and measurement tool (e.g., Nordic Musculoskeletal Questionnaire, Oswestry Disability Index, 10-point Likert scale, self-report).
- Prevalence data: Numerators and denominators for each reported time frame and subgroup (e.g., students vs. faculty, regions).
- Risk factor data: Effect estimates (e.g., ORs, relative risks) and corresponding 95% confidence intervals (CIs) for age, sex, smoking, family history of LBP, college seating or furniture, and other reported risk factors; where necessary, raw cross-tabulation data were extracted to calculate ORs.

Where information was missing or unclear, we inferred values from reported statistics (e.g., confidence intervals, *p*-values) using standard formulae; no imputation across studies was undertaken; however, no contact with authors was required for this review.

### 2.4. Assessment of Methodological Quality and Risk of Bias

The methodological quality and risk of bias of each included study were assessed using the Joanna Briggs Institute (JBI) checklist for analytical cross-sectional studies, which is appropriate for prevalence and risk factor designs [15]. Two reviewers independently evaluated each study across domains including:

- Clear definition and inclusion criteria for the target population.
- Appropriate measurement of exposure (risk factors) and outcome (LBP).
- Valid and reliable outcome measures.
- Identification and management of confounding factors.
- Appropriateness of statistical analysis.

Each item was rated as “yes”, “no”, “unclear”, or “not applicable” in line with JBI guidance, and an overall judgment of methodological quality (e.g., low, moderate, or high risk of bias) was derived for descriptive purposes; no arbitrary numerical cutoff was used to exclude studies. Disagreements were resolved by discussion, with the involvement of a third reviewer when necessary. The quality assessment informed the interpretation of findings and sensitivity analyses but did not serve as an exclusion criterion.

### 2.5. Data Synthesis and Statistical Analysis

All quantitative analyses were performed using a random effects meta-analytic approach to account for expected between-study heterogeneity in populations, measurement tools, and recall periods [13]. All meta-analyses were conducted using Review Manager (RevMan) version 5.4 (Cochrane Collaboration, London, UK).

#### 2.5.1. Prevalence Meta-Analysis

For each study, prevalence proportions of LBP were calculated as the number of participants with LBP divided by the total sample size for each relevant time frame (e.g., 1 week, 12 months, lifetime, at questionnaire, >3 months). Pooled prevalence estimates and corresponding 95% CIs were then derived using random effects models, stratified by:

- Timing: 1 week, 12 months, at time of questionnaire, >3 months, and lifetime prevalence.
- Population: Students vs. faculty and overall.
- Region: Eastern, Middle, Northern, Southern, Western, and overall, across regions.
- Measurement scale: Nordic Musculoskeletal Questionnaire, Oswestry Disability Index, 10-point Likert scale, self-report, and unclassified measures.

Between-study heterogeneity was quantified using the I^2^ statistic and τ^2^, with values of I^2^ >75% interpreted as indicating substantial heterogeneity. Cochran’s Q test was used to assess the statistical significance of heterogeneity. Subgroup analyses were conducted to explore whether prevalence differed systematically by timing, population group, region, or measurement tool, and tests for subgroup differences were performed (Q_b).

#### 2.5.2. Meta-Analysis of Risk Factors

For each risk factor (age, male gender, smoking, family history of LBP, and college seating/furniture), ORs and their standard errors were pooled using random effects models. Where multiple adjusted and unadjusted estimates were available, the most fully adjusted OR was preferred; otherwise, crude ORs were used. Pooled ORs with 95% CIs were calculated, and statistical significance was assessed using z tests.

Heterogeneity for each risk factor analysis was again assessed using I^2^, τ^2^, and Cochran’s Q. For example, age [14] yielded a pooled OR of 1.17 (95% CI: 1.01–1.34) with moderate heterogeneity (I^2^ ≈ 34%), whereas smoking (five studies) showed negligible heterogeneity (I^2^ = 0%) and a non-significant pooled OR of 1.03.

#### 2.5.3. Sensitivity Analyses

To examine the robustness of the pooled prevalence estimates, leave-one-out sensitivity analyses were conducted, sequentially omitting each study and recalculating the pooled prevalence. This procedure evaluated whether any single study exerted undue influence on overall estimates. In addition, exploratory comparisons were made between studies classified as higher versus lower methodological quality (based on JBI assessment) to assess whether study quality materially affected prevalence estimates. These analyses showed that exclusion of individual studies or restriction to higher-quality studies did not meaningfully alter the overall pooled prevalence, suggesting stable and robust findings.

Publication bias was not formally assessed using funnel plots or statistical tests (e.g., Egger’s test) due to the small number of studies in several subgroups and the substantial heterogeneity observed, which can compromise the interpretability of such methods. For the main prevalence synthesis, potential publication bias was examined using funnel plots, Egger’s regression and Begg–Mazumdar rank correlation tests, with trim-and-fill adjustment where appropriate.

In addition to quantitative pooling, we planned sensitivity analyses to explore the influence of study quality on the pooled prevalence and risk factor estimates. Specifically, where at least three studies contributed to a given meta-analysis, we repeated the analyses after excluding studies judged to be at high risk of bias on the JBI checklist to assess the robustness of the findings to study quality.

#### 2.5.4. Certainty of Evidence (GRADE) Assessment

In addition to the primary meta-analyses, we assessed the certainty of evidence for key outcomes using the Grading of Recommendations, Assessment, Development and Evaluation (GRADE) approach. For each outcome, we rated certainty across the domains of risk of bias, inconsistency, indirectness, imprecision, and publication bias. Given that all contributing studies had observational cross-sectional designs, the initial certainty level was set to ‘low’ and then downgraded where serious or very serious concerns were present. We applied GRADE to the pooled 12-month, point, and lifetime prevalence estimates of low back pain (LBP), as well as to pooled odds ratios (ORs) for the associations between age (per year) and LBP and between poor college seating and LBP. Final ratings (very low or low–very low) and the main reasons for downgrading were determined by consensus of the reviewers.

## 3. Results

### 3.1. Systematic Review Results

Table 1 summarizes 13 cross-sectional studies conducted across all five regions of Saudi Arabia, encompassing both university students and faculty with sample sizes ranging from 123 to 1163 participants. Most studies targeted young adult students, although a smaller number focused on middle-aged faculty, and a variety of LBP measurement tools were used, including the Oswestry Disability Index, Nordic Musculoskeletal Questionnaire, 10-point Likert scales, and self-report instruments. Reported prevalence estimates varied widely, with lifetime prevalence ranging from approximately one-third to over two-thirds and point or short-term prevalence spanning from 11% to 94%, reflecting substantial methodological and contextual heterogeneity. Several studies highlighted ergonomic and study-related factors, particularly poor college seating, prolonged sitting, and posture, while age and family history showed more modest or inconsistent associations across individual studies, in line with the pooled meta-analytic findings.

### 3.2. Risk of Bias Assessment

Risk of bias was assessed using the Joanna Briggs Institute (JBI) checklist for analytical cross-sectional studies, focusing on key domains such as sampling, measurement, and control of confounding. Overall, most included studies clearly defined their target population and applied explicit inclusion criteria, but several used convenience sampling and did not report response rates in sufficient detail, introducing potential selection bias. In terms of outcome assessment, low back pain was generally measured using validated tools such as the Oswestry Disability Index and the Nordic Musculoskeletal Questionnaire, although some studies relied on unclassified self-report instruments, which may have reduced measurement consistency. Important potential confounders—such as age, sex, physical activity, and psychosocial factors—were variably measured and only inconsistently adjusted for in multivariable analyses, raising concerns about residual confounding in the reported associations between risk factors and low back pain. Despite these limitations, no study was judged to have such a high risk of bias that exclusion was warranted; instead, the identified methodological concerns were considered when interpreting the pooled prevalence and risk factor estimates and partly explain the substantial heterogeneity observed across studies.

### 3.3. Meta-Analysis Results

#### 3.3.1. Prevalence Meta-Analysis

Across the included studies, LBP prevalence among university attendants in Saudi Arabia was high but varied markedly according to recall period, population group, region, and measurement tool. One-week prevalence ranged from 22% to 61%, with a pooled estimate of 39%, indicating that around two in five participants reported recent LBP, although heterogeneity between the three contributing studies was extreme. Over a 12-month period, prevalence estimates clustered between 33% and 61%, yielding a pooled prevalence of 42% and again showing very high heterogeneity, suggesting that methodological and contextual differences substantially influence yearly prevalence. Point prevalence at the time of questionnaire administration was higher, with individual estimates from 11% to 94% and a pooled estimate of 62%, implying that a substantial proportion of students and staff are symptomatic at any given time, though estimates differed widely between studies. In contrast, the prevalence of symptoms persisting more than three months was lower at 28%, reflecting the smaller subgroup of individuals with more chronic complaints. Lifetime prevalence ranged from 32% to 68%, with a pooled estimate of 55%, consistent with LBP being a common experience across the life course in university populations.

When all recall periods were combined, the overall pooled prevalence was 57%, but heterogeneity remained extremely high, and tests of subgroup differences confirmed that prevalence differed significantly across timing categories. In a separate model used for publication bias diagnostics, the unadjusted pooled proportion was 0.49 (95% CI 0.38–0.59), and after trim-and-fill, 0.44 (95% CI 0.32–0.56); these estimates were broadly consistent with the main analysis. Stratification by population showed that faculty members had a pooled prevalence of 50% and students 58%, with wide ranges in individual studies (32–69% in faculty, 11–94% in students) and no statistically significant difference between groups overall, suggesting that both groups were heavily affected. Region-specific analyses revealed pronounced geographic variation: the Eastern region exhibited the highest pooled prevalence (94%) with no heterogeneity; the Southern (67%) and Western (55%) regions showed intermediate values; the Middle region had a pooled prevalence of 50% with large between-study variation; and the Northern region showed the lowest prevalence at 27%. The overall pooled prevalence across all regions was 57%, and subgroup tests demonstrated highly significant regional differences, implying that local environmental, ergonomic, or cultural factors likely modulate LBP risk.

**Figure 2:**
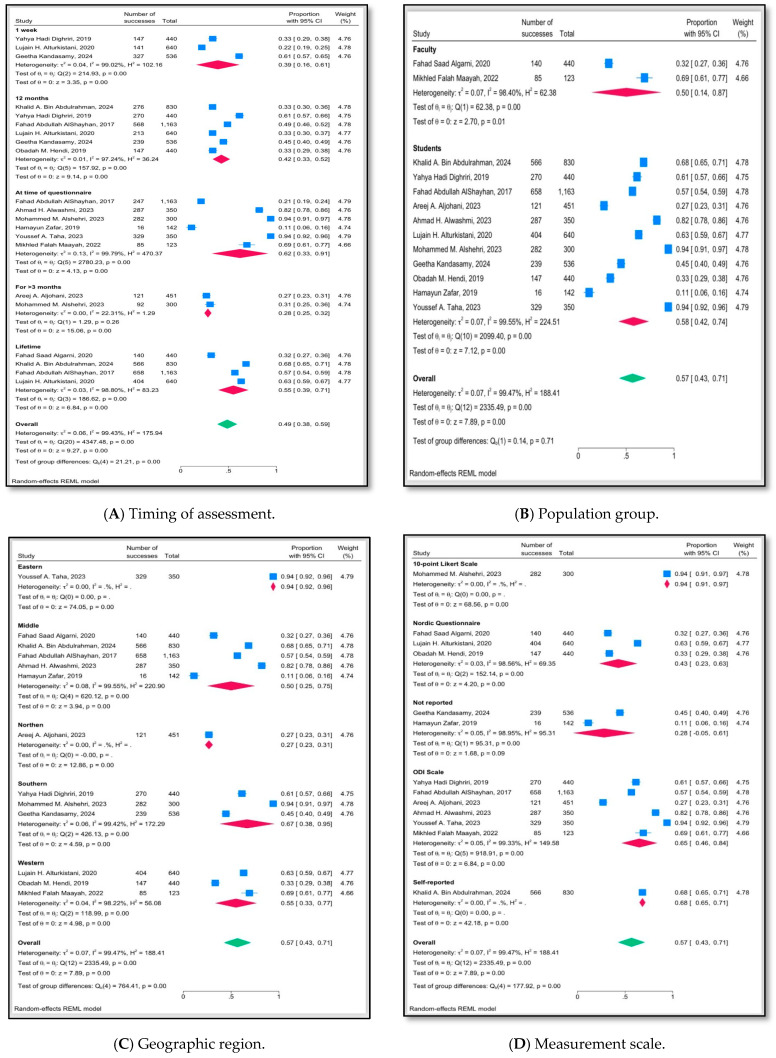
Forest plots of pooled prevalence of low back pain among university attendants in Saudi Arabia by (**A**) timing of assessment, (**B**) population group, (**C**) geographic region, and (**D**) measurement scale [10,11,16,17,18,19,20,21,22,23,24,25,26].

Measurement instruments also strongly influenced reported prevalence (Figure 2). A single study using a 10-point Likert scale reported a very high prevalence of 94%. Studies using the Nordic Musculoskeletal Questionnaire yielded a pooled prevalence of 43%, while those employing unclassified tools produced a lower and statistically non-significant pooled estimate of 28% with extreme heterogeneity. In contrast, six studies using the Oswestry Disability Index reported higher prevalence values (27–94%) and a pooled estimate of 65%, whereas one large study based on self-reported LBP status found a prevalence of 68%. Across all measurement scales, the pooled prevalence was 57%, but tests for subgroup differences showed that the choice of instruments significantly affected prevalence estimates, underlining the importance of standardized measurement. Overall, these findings indicate that while LBP is consistently common among university attendants in Saudi Arabia, its estimated prevalence is highly sensitive to recall period, target population, geographic setting, and, particularly, the measurement scale used.

#### 3.3.2. Risk Factor Meta-Analysis

This meta-analysis demonstrates that risk factors for LBP among university populations in Saudi Arabia show differing strengths and consistencies of association. Age exhibited a modest but statistically significant effect: pooling two studies yielded an OR of 1.17 (95% CI 1.01–1.34), with only moderate, non-significant heterogeneity, suggesting that increasing age is a reasonably consistent predictor of LBP in this setting. In contrast, male gender did not show a clear association; although one study reported more than double the odds of LBP in men, the overall pooled OR of 1.25 (95% CI 0.72–1.77) was non-significant, and heterogeneity was high, indicating that sex-related differences are inconsistent across studies.

**Figure 3:**
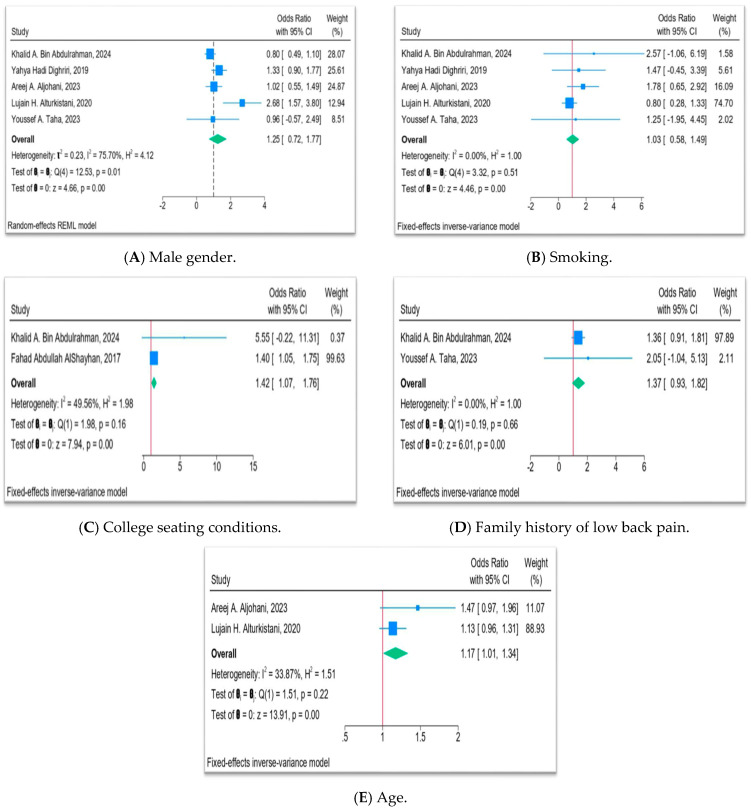
Forest plots of pooled effects of key risk factors for low back pain among university attendants in Saudi Arabia, including (**A**) male gender, (**B**) smoking, (**C**) college seating conditions, (**D**) family history of low back pain, and (**E**) age [11,16,18,20,23,25].

Smoking status likewise showed no meaningful relationship with LBP. Across five studies, the pooled OR was 1.03 (95% CI 0.58–1.49), with very low heterogeneity, implying that smoking is not an important independent determinant of LBP in these university cohorts. Family history of LBP showed a non-significant trend towards increased risk (pooled OR 1.37, 95% CI 0.93–1.82), but estimates were imprecise and based on only two studies, so hereditary or shared environmental influences remain uncertain. By contrast, college seating conditions emerged as the most robust modifiable factor: despite only two studies contributing data, the pooled OR of 1.42 (95% CI 1.07–1.76) indicated a statistically significant and practically relevant association between poor seating and higher odds of LBP, with moderate heterogeneity. However, this meta-analysis was based on data from only two studies, which limits the precision and reliability of this estimate and warrants cautious interpretation. Overall, these findings suggest that age and, particularly, suboptimal classroom furniture play a clearer role in LBP risk than gender, smoking, or reported family history in Saudi university settings (Figure 3).

### 3.4. Sensitivity Analysis

The sensitivity analysis was conducted to assess the robustness of the pooled prevalence estimates and examine the potential impact of individual studies on the overall results. This analysis involved systematically excluding one study at a time to determine whether any single study disproportionately influenced the pooled prevalence estimate. The results showed that the exclusion of specific studies did not significantly alter the overall pooled prevalence, indicating that the findings were robust and not highly sensitive to individual study data. Additionally, the influence of study quality was evaluated by comparing the results from studies with high and low methodological quality. Studies with higher quality generally reported slightly lower prevalence estimates, but the overall pooled prevalence remained stable. These findings suggest that the conclusions drawn from the meta-analysis are reliable, and the pooled prevalence estimates are not unduly affected by outliers, study quality, or individual study data, as shown in the figure.

**Figure 4:**
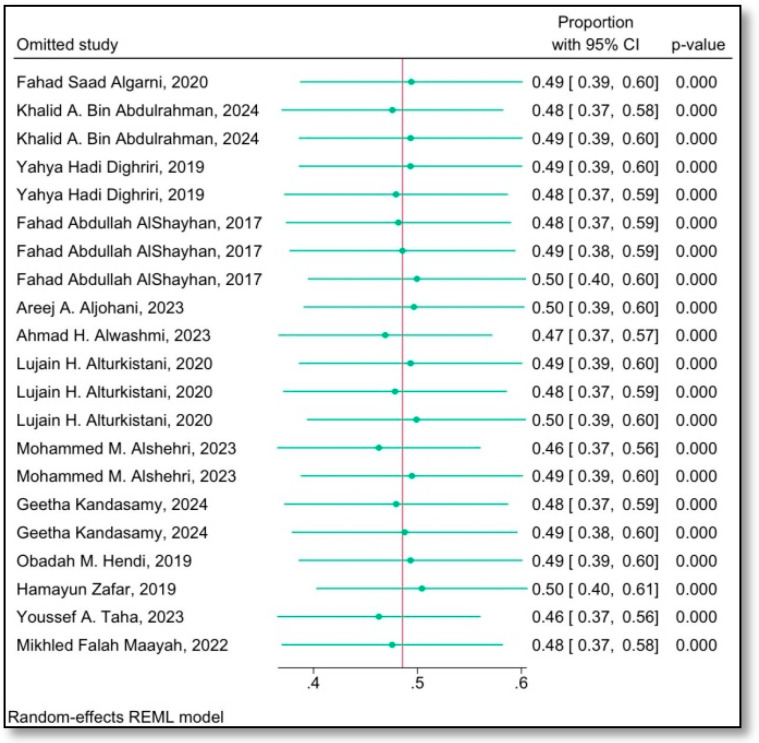
Leave-one-out sensitivity analysis for the pooled prevalence of low back pain among university attendants in Saudi Arabia [10,11,16,17,18,19,20,21,22,23,24,25,26].

The leave-one-out sensitivity analysis (Figure 4) evaluates how strongly each individual study affects the pooled prevalence estimate by removing one study at a time and recalculating the overall proportion. In this figure, the pooled prevalence and its 95% confidence interval remain very similar across all omissions, and all models stay statistically significant. This pattern indicates that no single study unduly influences the summary estimate and that the overall pooled prevalence of low back pain is robust and stable despite the high heterogeneity observed elsewhere in the meta-analysis.

**Figure 5:**
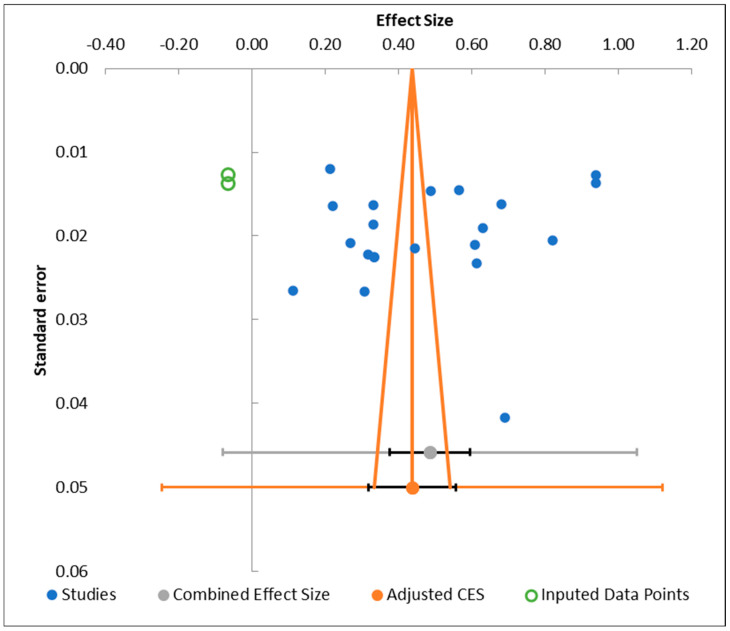
Funnel plot and quantitative tests assessing publication bias in the pooled prevalence of low back pain among university attendants in Saudi Arabia.

The publication bias diagnostics suggest that small-study or reporting bias is unlikely to materially distort the meta-analysis findings, although precision is limited by extreme heterogeneity. The unadjusted pooled proportion was 0.49 (95% CI 0.38–0.59), with very high heterogeneity (Q = 7655.22, *p* < 0.001; I^2^ = 99.71%), and a wide prediction interval from −0.08 to 1.05, indicating substantial between-study variability. After applying the trim-and-fill procedure, two potentially missing studies were imputed, yielding a slightly lower adjusted pooled estimate of 0.44 (95% CI 0.32–0.56) and a similarly wide prediction interval (−0.25 to 1.12), suggesting that any asymmetry would only modestly reduce the central effect size. Egger’s regression test showed a non-significant intercept (*p* = 0.899), and Begg and Mazumdar’s rank correlation test also did not reach significance (Kendall’s tau = −0.12, *p* = 0.225), both indicating no statistically demonstrable small-study effects. These results are visually supported by the funnel plot shown in Figure 5, which does not display marked asymmetry.

We conducted exploratory sensitivity analyses where data were restricted to studies judged to be at low or moderate risk of bias, which yielded pooled prevalence and risk factor estimates that were broadly similar to the main analyses, with overlapping confidence intervals and no systematic shift in effect direction. Although the wide confidence intervals and persistent heterogeneity limit precision, these findings suggest that inclusion of high-risk studies did not materially alter the central conclusions of the review.

### 3.5. Certainty of the Evidence (GRADE)

Using the GRADE framework, the certainty of evidence for all prevalence outcomes was judged to be very low. The pooled 12-month prevalence of LBP was 42% (95% CI 33–61), the point prevalence at the time of questionnaire was 62% (95% CI 49–75), and the lifetime prevalence was 55% (95% CI 41–69). Each of these estimates was downgraded for high risk of bias, because most included studies used non-probability sampling and incompletely reported response rates or control of confounding, and for very serious inconsistency, reflected in extreme between-study heterogeneity and, for 12-month prevalence, a wide prediction interval. As a result, the certainty that these pooled prevalence values closely approximate the true prevalence in the target population is very low.

For risk factor outcomes, the certainty of evidence was also limited. The pooled association between age and LBP (per additional year of age) yielded an OR of 1.17 (95% CI 1.01–1.34) and was rated as very low certainty, owing to the observational design, residual confounding, and imprecision associated with a relatively small number of contributing studies and a confidence interval close to the null. The association between poor college seating and LBP showed an OR of 1.42 (95% CI 1.07–1.76) and was judged to have low to very low certainty. Although this effect was statistically significant and reasonably precise, it was based on a small number of observational studies at risk of bias, particularly with respect to exposure assessment and incomplete adjustment for other ergonomic and psychosocial determinants (Table 2).

## 4. Discussion

This systematic review and meta-analysis aimed to estimate the pooled prevalence of LBP among university attendants in Saudi Arabia and to quantify its associations with key demographic and environmental risk factors. The pooled prevalence of LBP of approximately 57% among university attendants in Saudi Arabia indicates that more than one in two individuals in this setting experience LBP, underscoring a substantial musculoskeletal burden in the higher education sector. This estimate is higher than general adult prevalence figures reported for Saudi Arabia, where LBP has been estimated to affect between 18.8% and 53.5% of adults [5], and is broadly consistent with evidence that university students and academic staff constitute a high-risk group because of sustained sitting, high cognitive load, and limited physical activity [6,9]. The high pooled prevalence aligns with global and regional data from the Global Burden of Disease studies, which identify LBP as a leading cause of disability worldwide and report particularly high burdens in the Middle East and North Africa [1,2]. Taken together, these findings position LBP as a major occupational and educational health challenge for Saudi universities, with implications for student performance, staff productivity, and long-term musculoskeletal health.

The subgroup analyses by recall period reveal important temporal patterns. Across included studies, short-term (1-week) prevalence was lower (pooled 39%) than 12-month (42%) and lifetime prevalence (55%), while prevalence at the time of questionnaire administration reached 62%. This gradient is expected because longer recall windows capture more episodes of LBP over time; however, the very high point prevalence suggests that a large proportion of university attendants are symptomatic at any given moment. Similar recall-dependent patterns have been reported among school teachers and other occupational groups, where point prevalence tends to be lower than 12-month and lifetime estimates [7,8]. The extremely high heterogeneity observed across time-based subgroups (I^2^ often >97%) indicates that differences in measurement tools, case definitions, and sampling strategies contribute substantially to variability and limit simple comparison of prevalence across studies. Nevertheless, even the lowest pooled estimates in this review are high enough to warrant routine screening and prevention within university health services. Given the observed variability in prevalence estimates across different measurement instruments, future studies in this region would benefit from adopting standardized, validated tools for LBP assessment. Instruments such as the Nordic Musculoskeletal Questionnaire, which has been widely used in occupational and educational settings, could facilitate more comparable prevalence estimates and more reliable pooling of data in subsequent meta-analyses.

Marked geographic variation was also evident, with pooled prevalence ranging from 27% in the Northern region to 94% in the Eastern region and intermediate values in the Middle, Western, and Southern regions. These differences may reflect true contextual variation—such as differences in campus design, availability of ergonomic furniture, transportation patterns, and regional lifestyle factors—but they may also stem from methodological variability, including differing measurement tools, sampling frames, and response rates. For instance, Eastern-region studies relied on the Oswestry Disability Index and focused on health science students, who may have heavier clinical and academic workloads, while Northern-region data were limited to a single study with a narrower definition of chronic LBP (>3 months). Similar regional gradients in LBP burden have been reported across countries and within national health systems, often linked to urbanization, occupational structures, and access to preventive services [2,5]. The substantial heterogeneity between regions (Q_b(4) = 764.41, *p* < 0.001) suggests that national strategies in Saudi Arabia should incorporate region-specific ergonomic and health promotion initiatives rather than assuming a uniform risk profile across the country.

Differences in prevalence according to measurement tools further highlight the methodological sensitivity of LBP estimates. Studies using the Oswestry Disability Index (ODI) yielded a pooled prevalence of 65%, whereas those using the Nordic Musculoskeletal Questionnaire (NMQ) produced lower pooled estimates of 43%, and a single study using a 10-point Likert scale reported 94% prevalence. Self-report without a standardized instrument produced a lifetime prevalence of 68%, while unclassified tools yielded lower and more unstable estimates with wide confidence intervals. These findings mirror broader methodological literature showing that instruments differ in their thresholds for defining “case” LBP, recall periods, and emphasis on pain versus disability, all of which can systematically shift prevalence estimates [3,4]. The strong subgroup effect by measurement scale (Q_b(4) = 764.41, *p* < 0.001) underlines the need for future studies in Saudi universities to adopt validated and standardized tools, ideally harmonized across institutions, to enable more reliable surveillance and meta-analytic synthesis.

When stratified by population group, the pooled prevalence was 58% for students and 50% for faculty, with no statistically significant difference between these categories. This suggests that both students and academic staff are heavily affected, although the higher point estimates in some faculty cohorts (e.g., up to 69%) may reflect cumulative exposure to occupational risk factors over longer careers [10,24]. Previous reviews in healthcare workers and teachers have also reported high LBP prevalence in professional groups with prolonged standing or sitting, manual handling, or psychosocial stress, supporting the notion that academic work environments are intrinsically demanding for the lumbar spine [5,7,8]. Importantly, the absence of a clear difference between students and faculty in the pooled analysis may be partly driven by high heterogeneity and overlapping contextual exposures—such as shared lecture halls and office spaces—rather than true equivalence of risk.

The risk factor meta-analysis provides nuanced insights into determinants of LBP within university settings. Age showed a modest but statistically significant pooled association with LBP (OR 1.17, 95% CI 1.01–1.34), based on two studies [23,25]. This finding is consistent with broader epidemiological evidence that LBP risk increases with age due to degenerative spinal changes, cumulative mechanical loading, and age-related declines in muscle strength and flexibility [1,3]. Within the university context, older students (e.g., postgraduate or part-time learners) and long-serving faculty may accumulate longer exposure to sedentary work patterns and poor ergonomics, which could partly explain the observed age gradient.

In contrast, male gender and smoking did not show significant pooled associations with LBP (OR 1.25 and 1.03, respectively, both with CIs crossing unity), despite some individual studies reporting significant effects. For example, Alturkistani [25] found higher odds of LBP among male students (OR 2.68), while other studies showed null or reversed associations, leading to substantial heterogeneity (I^2^ = 75.7% for gender). These inconsistencies echo findings in other populations, where sex-specific LBP patterns often depend on the interplay of occupational roles, pain reporting behaviors, and cultural norms [6,8]. Similarly, although smoking has been proposed as a biological and behavioral risk factor for spinal pain, the pooled null association in this review and the absence of heterogeneity (I^2^ = 0%) suggest that smoking is not a major independent driver of LBP in Saudi university settings, or that its effects are overshadowed by stronger mechanical and psychosocial factors.

Family history of LBP showed a trend towards increased risk (pooled OR 1.37, 95% CI 0.93–1.82) without reaching statistical significance. This pattern is compatible with evidence that genetic predisposition and shared lifestyle or ergonomic habits within families can contribute to LBP, but the small number of available studies and wide confidence intervals preclude firm conclusions [1,2]. Future research with larger, well-characterized samples could clarify the relative contributions of hereditary versus environmental influences on LBP in young adults.

Among the examined risk factors, college seating conditions emerged as the robust predictor, with a pooled OR of 1.42 (95% CI 1.07–1.76). Studies by AlShayhan [16] and Bin Abdulrahman [11] consistently showed that uncomfortable, non-ergonomic seats and desks were associated with higher odds of LBP, even when accounting for other variables. This finding aligns with international literature linking poor classroom ergonomics, prolonged static sitting, and inadequate back support to increased LBP among students and office workers [6,7,9]. The strength and consistency of this association across Saudi studies highlight ergonomic modification of lecture halls, laboratories, and office spaces as a prime target for low-cost, high-impact interventions in universities.

The high I^2^ statistics (>99% in many pooled analyses) highlight substantial heterogeneity across studies, which is a key limitation but also an informative result. Sources of heterogeneity likely include differences in measurement tools, recall periods, academic disciplines, campus infrastructure, and sampling strategies, as well as unmeasured factors such as physical activity, psychosocial stress, and comorbidities. Similar levels of heterogeneity have been reported in meta-analyses of LBP among school teachers and health workers, where diverse settings and methodological approaches complicate synthesis [5,8]. The leave-one-out sensitivity analysis in this review showed that the pooled prevalence remained stable when excluding individual studies, suggesting that the overall burden estimate is robust despite heterogeneity; however, the wide prediction intervals implied by such variability mean that the actual prevalence in any single university could be much higher or lower than the pooled value. This reinforces the importance of local surveillance and tailored interventions alongside national and regional policies.

The GRADE assessment indicates that the certainty of evidence underlying our key findings is generally very low. Although the pooled prevalence estimates (42% for 12-month, 62% for point, and 55% for lifetime LBP) highlight a substantial burden among university attendants, confidence in these figures is reduced by high risk of bias in the primary studies and extreme inconsistency across samples, regions, and measurement tools, leading to very low certainty ratings. Increasing age and poor college seating conditions were significantly associated with LBP, whereas male gender, smoking, and family history showed non-significant pooled effects; notably, seating ergonomics represent a modifiable, actionable risk factor in contrast to non-modifiable determinants such as age, underscoring the potential impact of targeted ergonomic interventions within Saudi universities. These GRADE ratings mean that the true prevalence and strength of associations may differ substantially from the pooled estimates, and they underscore the need for future, well-designed longitudinal and interventional studies using representative sampling, standardized LBP measures, and rigorous control of confounding. The methodological rigor of this review, including adherence to Cochrane and PRISMA 2020 guidance and prospective PROSPERO registration, provides a robust baseline against which future longitudinal and intervention studies in the region can be benchmarked. The substantial between-study heterogeneity is likely multifactorial. Differences in LBP case definitions and recall periods, variability in measurement tools (including use or non-use of validated questionnaires), and heterogeneity in university environments and regional academic cultures (e.g., timetable structures, classroom ergonomics, and norms around physical activity) may each contribute to divergent prevalence estimates. These contextual and methodological variations reduce comparability across studies and were a key reason for downgrading the certainty of the prevalence evidence in our GRADE assessment.

Several methodological limitations of the included studies further temper the interpretation of the results. Many relied on convenience sampling and did not report response rates, which raises concerns about selection bias, especially if students or staff with LBP are more likely to participate. Demographic and behavioral covariates (e.g., body mass index, physical activity, psychosocial stress) were not consistently measured or adjusted for, increasing the risk of residual confounding in risk factor analyses. Additionally, the cross-sectional design of all included studies precludes causal inference; associations between seating conditions, age, and LBP may be bidirectional or influenced by unmeasured variables. These weaknesses are comparable to those noted in other occupational LBP reviews and underscore the need for high-quality longitudinal and intervention studies in university settings [5,7]. Review processes were limited by restriction to English-language publications and to three major databases, and we did not contact study authors for additional data.

Despite these limitations, this systematic review and meta-analysis contributes important evidence specific to Saudi universities, filling a gap left by prior national and international reviews that either focused on other occupational groups or aggregated very heterogeneous educational settings [5,8]. The findings support the prioritization of ergonomic interventions—particularly improved seating and workstations—alongside educational and behavioral strategies to promote regular movement, posture awareness, and physical activity among both students and staff. From a rehabilitation perspective, university-based physiotherapy and occupational health services could play a central role in screening for early LBP, providing targeted exercise and education programs, and advising on ergonomic redesign of classrooms and offices.

## 5. Conclusions

This systematic review and meta-analysis demonstrate that LBP is a pervasive health problem among university attendants in Saudi Arabia, with pooled prevalence estimates exceeding those reported for the general adult population and comparable high-risk occupational groups. The consistently elevated prevalence across regions and recall periods, together with the strong association with suboptimal seating and the modest but significant impact of age, underscores the central role of ergonomic and age-sensitive strategies in mitigating LBP in higher education environments; however, these cross-sectional data cannot establish temporal or causal relationships, and the findings should therefore be interpreted as hypothesis-generating. Conversely, the non-significant pooled effects of male gender, smoking, and family history suggest that demographic and lifestyle factors may be less critical than mechanical and organizational exposures within universities. Given the very high heterogeneity across studies, future research should prioritize standardized LBP definitions, validated measurement tools, and longitudinal designs to clarify causal pathways and evaluate targeted interventions. For practice and policy, universities in Saudi Arabia should consider integrating ergonomics-informed infrastructure upgrades, posture and activity education, and accessible rehabilitation services as core components of student and staff health programs. To enhance comparability and strengthen future evidence syntheses, we recommend that researchers in Saudi universities adopt standardized LBP measurement instruments such as the Nordic Musculoskeletal Questionnaire and report methods transparently alongside longitudinal designs. Prospective cohort and intervention studies are needed to test whether modifications to seating ergonomics and other contextual factors lead to measurable reductions in LBP incidence and persistence among university attendants.

## Figures and Tables

**Table 1:** Characteristics and main findings of studies included in the systematic review on low back pain among university attendants (students and staff) in Saudi Arabia.

First Author (Year)	Region (SA)	Population	Sample Size (*n*)	Mean Age (Years)	LBP Definition and Measurement Tool	Recall Period(s) Reported	LBP Prevalence	Other Prevalence Estimates	Key Risk Factors Reported
AlShayhan [16]	Eastern and Middle	Students in Saudi Arabia	1163	Young adults	Low back pain assessed using Oswestry Disability Index (ODI)	12-month, point (at questionnaire), lifetime	57% lifetime prevalence (568/1163)	21% at time of questionnaire (247/1163)	Poor college seating/furniture associated with higher odds of LBP (significant); male gender not consistently associated
Algarni [10]	Middle and Western	Students in Saudi Arabia	440	Middle-aged adults	Nordic Musculoskeletal Questionnaire (NMQ)	Lifetime	32% lifetime prevalence (140/440)	Not reported for shorter recall	Faculty occupational factors and seating conditions associated with LBP; age trend suggested but not quantified in pooled model
Zafar [17]	Middle	Students in Saudi Arabia	142	Young adults	Unclassified self-report instrument	At time of questionnaire	11% point prevalence (16/142)	Not reported for other time frames	No consistent significant associations for sex or smoking when pooled; local study suggests contribution of prolonged sitting
Dighriri [18]	Southern	Students in Saudi Arabia	440	Young adults	Oswestry Disability Index (ODI)	1-week, 12-month	61% 12-month prevalence (270/440)	33% 1-week prevalence (147/440)	Prolonged sitting and poor posture associated with LBP; gender and smoking not significant in pooled analysis
Hendi [19]	Western	Students in Saudi Arabia	440	Young adults	Nordic Musculoskeletal Questionnaire (NMQ)	12-month	33% 12-month prevalence (147/440)	Not reported for lifetime	Ergonomic and study-related factors highlighted; no consistent independent effect for smoking in pooled estimates
Taha [20]	Eastern	Students in Saudi Arabia	350	Young adults	Oswestry Disability Index (ODI)	At time of questionnaire	94% point prevalence (329/350)	Not reported for other recall periods	Family history of LBP shows possible association (pooled OR 1.37, not statistically significant); seating remains important predictor
Alwashmi [21]	Middle	Students in Saudi Arabia	350	Young adults	Oswestry Disability Index (ODI)	At time of questionnaire	82% point prevalence (287/350)	12-month prevalence not consistently reported	Higher LBP prevalence associated with poor ergonomics and prolonged sitting; smoking not significant in pooled model
Alshehri [22]	Southern	Students in Saudi Arabia	300	Young adults	10-point Likert scale for LBP severity/frequency	At time of questionnaire, >3 months	94% point prevalence (282/300)	31% prevalence >3 months (92/300)	Longer symptom duration associated with older age; measurement tool contributed to higher prevalence compared with NMQ
Aljohani [23]	Northern	Students in Saudi Arabia	451	Young adults	Oswestry Disability Index (ODI)	>3 months	27% prevalence >3 months (121/451)	Not reported for point or 12-month prevalence	Age showed increased odds of LBP (OR 1.47, 95% CI 0.97–1.96) contributing to pooled age effect (OR 1.17)
Maayah [24]	Western	Faculty in Saudi Arabia	123	Middle-aged adults	Oswestry Disability Index (ODI)	At time of questionnaire	69% point prevalence (85/123)	Not reported for other time frames	Faculty role and ergonomic factors associated with LBP; region contributes to between-study heterogeneity
Alturkistani [25]	Middle and Western	Students in Saudi Arabia	640	Young adults	Nordic Musculoskeletal Questionnaire (NMQ)	1-week, 12-month, lifetime	33% 12-month prevalence (213/640)	22% 1-week (141/640); 63% lifetime (404/640)	Age (OR 1.13, 95% CI 0.96–1.31) and male gender (OR 2.68, 95% CI 1.57–3.80) showed significant associations in single-study analysis; seating also implicated
Bin Abdulrahman [11]	Middle	Students in Saudi Arabia	830	Young adults	Self-reported LBP status	12-month, lifetime	33% 12-month prevalence (276/830)	68% lifetime prevalence (566/830)	Family history of LBP (OR 1.36, 95% CI 0.91–1.81) and poor seating/furniture associated with higher LBP odds (pooled OR for seats 1.42)
Kandasamy [26]	Southern	Students in Saudi Arabia	536	Young adults	Unclassified questionnaire for LBP	1-week, 12-month	45% 12-month prevalence (239/536)	61% 1-week prevalence (327/536)	Regional and ergonomic factors emphasized; contributes to high heterogeneity within Southern region subgroup

**Table 2:** Summary of pooled effects and certainty of evidence (GRADE) for low back pain prevalence and risk factor outcomes among university attendants in Saudi Arabia (search from inception to February 2025).

Outcome	Effect (Pooled Result)	Certainty (GRADE)	Main Reasons for Rating
12-month LBP prevalence	42% (95% CI 33–61)	Very low	High risk of bias; extreme heterogeneity; wide prediction interval
Point prevalence (at questionnaire)	62% (95% CI 49–75)	Very low	High risk of bias; extreme heterogeneity
Lifetime prevalence	55% (95% CI 41–69)	Very low	High risk of bias; inconsistency across tools and regions
Age (per year) and LBP	OR 1.17 (95% CI 1.01–1.34)	Very low	Observational design; residual confounding; imprecision
Poor seating and LBP	OR 1.42 (95% CI 1.07–1.76)	Low–very low	Observational design; risk of bias; effect reasonably precise

## Data Availability

All data underlying the findings of this systematic review and meta-analysis were extracted from published articles that are cited in the reference list. Extracted datasets and analytic code are available from the corresponding author upon reasonable request.

## Supplementary Materials

The following supporting information can be downloaded at: https://www.mdpi.com/article/10.3390/jcm15072808/s1, File S1. PRISMA_2020_checklist. File S2. PRIS-MA_2020_abstract_checklist.

## Abbreviations

The following abbreviations are used in this manuscript:

LBP	Low back pain
OR	Odds ratio
CI	Confidence interval
PRISMA	Preferred Reporting Items for Systematic Reviews and Meta-Analyses
JBI	Joanna Briggs Institute
NMQ	Nordic Musculoskeletal Questionnaire
ODI	Oswestry Disability Index
PROSPERO	International Prospective Register of Systematic Reviews
