# Supplementary material for: Mapping the Prevalence and Risk Factors of Low Back Pain Among University Populations in Saudi Arabia: A Systematic Review and Meta-Analysis
Source: J Clin Med. 2026 Apr 7;15(7):2808. doi: 10.3390/jcm15072808 (PMC13073772; doi:10.3390/jcm15072808)
Supplement: Supplementary file 1 [file jcm-15-02808-s001.zip › PRISMA checklist/PRISMA_2020_checklist 1.pdf]

PRISMA 2020 Checklist

Section and Topic	Item #	Checklist item	Location where item is reported
**TITLE**			
Title	1	Identify the report as a systematic review.	Title page ("…A Systematic Review and Meta-Analysis")
**ABSTRACT**			
Abstract	2	See the PRISMA 2020 for Abstracts checklist.	Structured abstract (Background/Objectives, Methods, Results, Conclusions).
**INTRODUCTION**			
Rationale	3	Describe the rationale for the review in the context of existing knowledge.	Introduction, first three paragraphs (global and regional LBP burden, Saudi context, university setting).
Objectives	4	Provide an explicit statement of the objective(s) or question(s) the review addresses.	Introduction, final paragraph ("The primary objective was… The secondary objective was…").
**METHODS**			
Eligibility criteria	5	Specify the inclusion and exclusion criteria for the review and how studies were grouped for the syntheses.	Materials and Methods, "Eligibility criteria" (2.2.1–2.2.6) and "Prevalence meta-analysis" (stratification).
Information sources	6	Specify all databases, registers, websites, organisations, reference lists and other sources searched or consulted to identify studies. Specify the date when each source was last searched or consulted.	2.3 Information sources and search strategy.
Search strategy	7	Present the full search strategies for all databases, registers and websites, including any filters and limits used.	2.3 Information sources and search strategy.
Selection process	8	Specify the methods used to decide whether a study met the inclusion criteria of the review, including how many reviewers screened each record and each report retrieved, whether they worked independently, and if applicable, details of automation tools used in the process.	2.3.1–2.3.3 Study selection (use of Covidence, two independent reviewers, third reviewer if needed, PRISMA flow).
Data collection process	9	Specify the methods used to collect data from reports, including how many reviewers collected data from each report, whether they worked independently, any processes for obtaining or confirming data from study investigators, and if applicable, details of automation tools used in the process.	2.3.4 Data extraction (two independent reviewers, piloted form, Covidence, no author contact).
Data items	10a	List and define all outcomes for which data were sought. Specify whether all results that were compatible with each outcome domain in each study were sought (e.g. for all measures, time points, analyses), and if not, the methods used to decide which results to collect.	2.2.3 Outcomes and 2.3.4 Data extraction (LBP prevalence by recall period; ORs for risk factors).
	10b	List and define all other variables for which data were sought (e.g. participant and intervention characteristics, funding sources). Describe any assumptions made about any missing or unclear information.	2.3.4 Data extraction (study and population characteristics, tools, risk factors; inferring values when unclear).
Study risk of bias assessment	11	Specify the methods used to assess risk of bias in the included studies, including details of the tool(s) used, how many reviewers assessed each study and whether they worked independently, and if applicable, details of automation tools used in the process.	2.4 Assessment of methodological quality and risk of bias (JBI checklist; two independent reviewers; resolution of disagreements).
Effect measures	12	Specify for each outcome the effect measure(s) (e.g. risk ratio, mean difference) used in the synthesis or presentation of results.	2.5 Data synthesis and statistical analysis, 2.5.1 and 2.5.2 (prevalence proportions with 95% CIs; ORs with 95% CIs).
Synthesis methods	13a	Describe the processes used to decide which studies were eligible for each synthesis (e.g. tabulating the study intervention characteristics and comparing against the planned groups for each synthesis (item #5)).	2.5.1 Prevalence meta-analysis (grouping by recall period, population, region, measurement scale; overall pooled).
	13b	Describe any methods required to prepare the data for presentation or synthesis, such as handling of	2.3.4 Data extraction and 2.5 Data synthesis

PRISMA 2020 Checklist

Section and Topic	Item #	Checklist item	Location where item is reported
		missing summary statistics, or data conversions.	(calculation of proportions and ORs, derivation from raw tables).
	13c	Describe any methods used to tabulate or visually display results of individual studies and syntheses.	2.5 Data synthesis (reference to forest plots) and Results (Table 1; Figs. 2–3).
	13d	Describe any methods used to synthesize results and provide a rationale for the choice(s). If meta-analysis was performed, describe the model(s), method(s) to identify the presence and extent of statistical heterogeneity, and software package(s) used.	2.5 Data synthesis and statistical analysis.
	13e	Describe any methods used to explore possible causes of heterogeneity among study results (e.g. subgroup analysis, meta-regression).	2.5.1 Prevalence meta-analysis (subgroups by timing, population, region, scale; Q_b tests).
	13f	Describe any sensitivity analyses conducted to assess robustness of the synthesized results.	2.5.3 Sensitivity analyses
Reporting bias assessment	14	Describe any methods used to assess risk of bias due to missing results in a synthesis (arising from reporting biases).	Methods (sentence at the end of 2.5.3) and Results (publication bias paragraph, Fig. 6).
Certainty assessment	15	Describe any methods used to assess certainty (or confidence) in the body of evidence for an outcome.	2.5.4 Certainty of evidence (GRADE) Assessment
**RESULTS**			
Study selection	16a	Describe the results of the search and selection process, from the number of records identified in the search to the number of studies included in the review, ideally using a flow diagram.	3.1 Systematic review results and Fig. 1 PRISMA flow diagram.
	16b	Cite studies that might appear to meet the inclusion criteria, but which were excluded, and explain why they were excluded.	Partly present (reasons documented, but list not shown).
Study characteristics	17	Cite each included study and present its characteristics.	3.1 Systematic review results and Table 1.
Risk of bias in studies	18	Present assessments of risk of bias for each included study.	3.2 Risk of bias assessment.
Results of individual studies	19	For all outcomes, present, for each study: (a) summary statistics for each group (where appropriate) and (b) an effect estimate and its precision (e.g. confidence/credible interval), ideally using structured tables or plots.	Table 1; forest plots in Figs. 2–3.
Results of syntheses	20a	For each synthesis, briefly summarise the characteristics and risk of bias among contributing studies.	3.3.1 Prevalence meta-analysis and 3.3.2 Risk factors meta-analysis (first paragraphs).
	20b	Present results of all statistical syntheses conducted. If meta-analysis was done, present for each the summary estimate and its precision (e.g. confidence/credible interval) and measures of statistical heterogeneity. If comparing groups, describe the direction of the effect.	3.3.1, 3.3.2 and Figs. 2–3 (pooled estimates, CIs, I^2, τ^2; direction of association).
	20c	Present results of all investigations of possible causes of heterogeneity among study results.	3.3.1 (subgroups by timing, region, population, measurement scale) and 3.3.2 (differences across risk factors).
	20d	Present results of all sensitivity analyses conducted to assess the robustness of the synthesized results.	3.4 Sensitivity analysis and Fig. 4.
Reporting biases	21	Present assessments of risk of bias due to missing results (arising from reporting biases) for each synthesis assessed.	Results section titled “Publication bias” or within 3.4 (ensure it explicitly references Fig. 6).
Certainty of evidence	22	Present assessments of certainty (or confidence) in the body of evidence for each outcome assessed.	*3.5 Certainty of the evidence (GRADE):*

PRISMA 2020 Checklist

Section and Topic	Item #	Checklist item	Location where item is reported
**DISCUSSION**			
Discussion	23a	Provide a general interpretation of the results in the context of other evidence.	Discussion, first paragraphs.
	23b	Discuss any limitations of the evidence included in the review.	Discussion, “Limitations” paragraph (heterogeneity, cross-sectional designs, self-report, residual confounding).
	23c	Discuss any limitations of the review processes used.	Discussion, “Limitations” paragraph (heterogeneity, cross-sectional designs, self-report, residual confounding).
	23d	Discuss implications of the results for practice, policy, and future research.	Discussion final paragraphs and Conclusions (ergonomic redesign, screening, need for longitudinal/interventional work).
**OTHER INFORMATION**			
Registration and protocol	24a	Provide registration information for the review, including register name and registration number, or state that the review was not registered.	Abstract Methods and 2.1 Protocol and registration (PROSPERO CRD420250654048).
	24b	Indicate where the review protocol can be accessed, or state that a protocol was not prepared.	The registered protocol, including full methods, is available in the PROSPERO record (CRD420250654048).
	24c	Describe and explain any amendments to information provided at registration or in the protocol.	2.1 Protocol and registration
Support	25	Describe sources of financial or non-financial support for the review, and the role of the funders or sponsors in the review.	Funding section
Competing interests	26	Declare any competing interests of review authors.	Conflicts of Interest statement
Availability of data, code and other materials	27	Report which of the following are publicly available and where they can be found: template data collection forms; data extracted from included studies; data used for all analyses; analytic code; any other materials used in the review.	Data Availability Statement

From: Page MJ, McKenzie JE, Bossuyt PM, Boutron I, Hoffmann TC, Mulrow CD, et al. The PRISMA 2020 statement: an updated guideline for reporting systematic reviews. BMJ 2021;372:n71. doi: 10.1136/bmj.n71. This work is licensed under CC BY 4.0. To view a copy of this license, visit https://creativecommons.org/licenses/by/4.0/
